# Shortened key growth periods of soybean observed in China under climate change

**DOI:** 10.1038/s41598-021-87618-9

**Published:** 2021-04-14

**Authors:** Qinghua Tan, Yujie Liu, Liang Dai, Tao Pan

**Affiliations:** 1 Key Laboratory of Land Surface Pattern and Simulation, Institute of Geographic Sciences and Natural Resources Research, Chinese Academy of Sciences, Beijing, 100101 China; 2 University of Chinese Academy of Sciences, Beijing, 100049 China

**Keywords:** Climate-change impacts, Climate change

## Abstract

Phenology is an important indicator of global climate change. Revealing the spatiotemporal characteristics of crop phenology is vital for ameliorating the adverse effects of climate change and guiding regional agricultural production. This study evaluated the spatiotemporal variability of soybean’s phenological stages and key growth periods, and assessed their sensitivity to key climatic factors, utilizing a long-term dataset (1992–2018) of soybean phenology and associated meteorological data collected at 51 stations across China. The results showed that (1) during the soybean growing seasons from 1992 to 2018, the average temperature (0.34 ± 0.09 ℃ decade^−1^) and cumulative precipitation (6.66 ± 0.93 mm decade^−1^) increased, but cumulative sunshine hours (− 33.98 ± 1.05 h decade^−1^) decreased. (2) On a national scale, dates of sowing, emergence, trifoliate, anthesis, and podding of soybean were delayed, while the maturity date showed an advancing trend. The vegetative growth period (− 0.52 ± 0.24 days decade^−1^) and whole growth period (− 1.32 ± 0.30 days decade^−1^) of soybean were shortened, but the reproductive growth period (0.05 ± 0.26 days decade^−1^) was slightly extended. Trends in soybean phenological stages and key growth periods diverged in regions. Soybean phenological stages were delayed in Huang-Huai-Hai soybean zone, whereas advanced in southern soybean zone. Moreover, the key growth periods were greatly shortened in northern soybean zone. (3) In general, the sensitivity of soybean key growth periods to temperature was negative, whereas those to precipitation and sunshine hours differed among regions. In particular, most phenological stages were negatively sensitive to sunshine hours. Our results will provide scientific support for decision-making in agricultural production practices.

## Introduction

Plant phenology is a cyclical development process of vegetation driven by climate environment, and it consequently has a high response to climate change, which also makes it a robust and valuable biological indicator of climate change^1–4^. In recent decades, crop phenology has been significantly affected by climate change, characterized as a remarkable climate warming^5,6^. Changes in local environmental conditions during crop growing season, affect the physiological processes and growth period duration of crop, and ultimately alter crop yields^7–10^. Therefore, understanding the changes in crop phenology is of great value for adapting to climate change and ensuring food security.

Research on crop phenology under the background of climate change indicates that different climatic factors may have distinct impacts on crop phenology^11,12^. Increased temperature can accelerate the growth and development of crops, thus shortening the time required for transition between key growth stages^13–15^, which is the main factor that influences crop phenological changes over time^16^. Meanwhile, variation in precipitation and sunshine hours can also affect crops’ phenological changes. For instance, as the precipitation increases, the key growth periods of maize were prolonged^17^, while the growing season length of wheat increased with the increasing of precipitation and sunshine duration during the growing season^18^. In addition, changes in the growth period of crops will also prompt farmers to adjust management measures, leading to changes in climatic conditions for crop growth, which will further affect crop phenology^19^.

Phenology of same crop significantly varied among different regions owing to the influence of local climate changes and management measures^18,20,21^. For instance, the sowing date (SD) and silking date of maize were advanced whereas its maturity date was delayed, and both vegetative and reproductive growth period were prolonged in the United States^22^. Abbas et al.^23^ showed that the SD and maturity of spring maize in Pakistan was advanced by 4.6 and 9.2 days decade^−1^, respectively. In China, Liu et al.^21^ found that the phenological phases of spring maize were mostly advanced, and the change of whole growth period varied in different cultivation regions. Spatial discrepancies were also detected in phenology changes of wheat^18,24^ and rice^21,25^. For soybean, its SD, anthesis date and maturity date were advanced, and the vegetative and whole growth periods were prolonged while the reproductive growth period has remained basically unchanged in United States^22^. Compared with the 1950s, the soybean growth period has been lengthened by 2.7 days decade^−1^ in Northeast China^26^.

Soybean is one of the main crops grown in China, the world’s largest consumer of soybeans. However, due to the rapid economic and social development, its domestic production cannot meet the growing demand^27^. The government encourages farmers to plant more soybean crops to achieve the overall soybean self-sufficiency in China. As the growth and yield of soybeans are greatly affected by climate change, it is important to understand the impact of climate change on soybean phenology. However, existing research on how climate change affect crop growth and production mainly focused on rice, wheat and maize, which are the three major food crops grown in China^6^. The impacts of climatic factors on the growth and production of soybean, especially its phenology, have not yet been fully studied. In addition, temperature, precipitation, and sunshine hours are the most important climatic factors to meet the demand of water, light, and heat resource for crop growth^17^. Previous research has emphasized the effects of temperature^5,11,28^, whereas the impact of other important climatic factors especially precipitation and sunshine hours upon soybean growth periods remain largely unknown^12,18,29^, which can increase uncertainty of results. Moreover, studies of temperature’s effect on soybean growth and yield were mainly performed at a local scale^30,31^, while less attention has been given to the spatiotemporal variation in changes of soybean phenology and its responses to climate change among different cultivation regions, which is critical to guide regional climate adaptation strategies and estimate the spatial heterogeneity of soybean production.

To fill this knowledge gap, using historical observation data (1992–2018) of soybean phenology from 51 agro-meteorological stations across China, we aims to: (1) analyze the trends of key climatic factors throughout the soybean growing season; (2) investigate the spatiotemporal variation of trends in soybean phenological stages and key growth periods under the background of climate change; and (3) explore the sensitivity of soybean phenological stages and key growth periods to different climatic factors.

## Results

### Spatiotemporal changes in climatic factors during the soybean growing season

**Figure 1:**
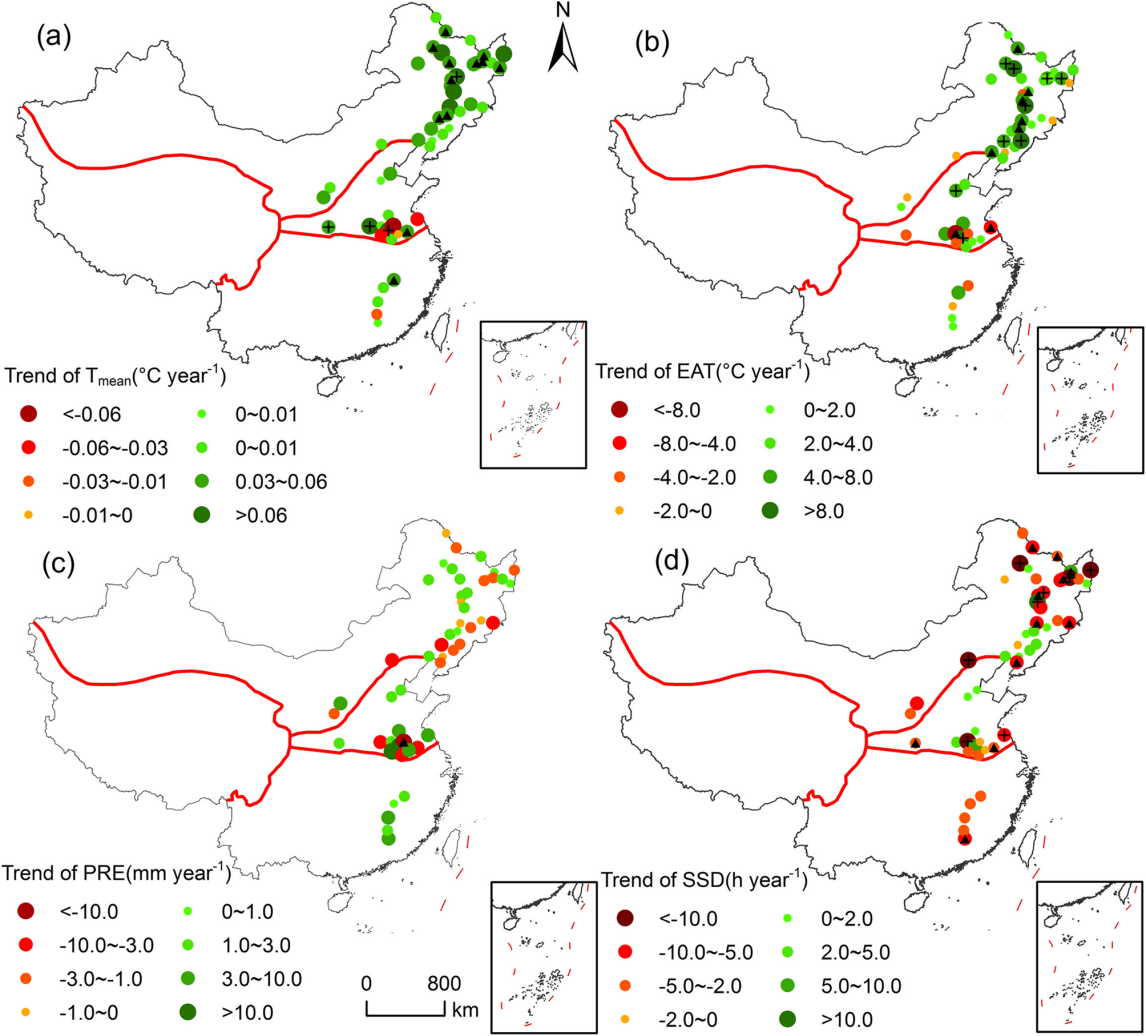
The trend of climatic factors during soybean growing seasons between 1992 and 2018. Abbreviations: ‘▲’, significant at the 0.05 level; ‘ + ’, significant at the 0.01 level. T_mean_, EAT, PRE, and SSD represent mean temperature, effective accumulated temperature, cumulative precipitation and sunshine hours, respectively. The map was generated by ArcGIS 10.2 software (https://www.esri.com/).

The trends of temperature, effective accumulated temperature (EAT), cumulative precipitation and sunshine hours during soybean growing seasons from 1992 to 2018 varied in stations (Fig. 1). Nationally, the average temperature during soybean growing season increased by 0.34 ± 0.09 ℃ decade^−1^ (92.15% of stations), and the probability of increasing average temperature was 80.59%. Regionally, the change rate of average temperature in northern soybean zone (0.38 ± 0.10 ℃ decade^−1^) was greater than that in summer soybean zone (0.12 ± 0.18 ℃ decade^−1^) and southern soybean zone (0.15 ± 0.25 ℃ decade^−1^). Especially with the increase of latitude, the warming trend of the northern spring soybean station has increased. Meanwhile, change trend of EAT during soybean growing season was consistent with average temperature, with median increase rate of 26.31 ± 1.21 ℃ decade^−1^. The probability of increase in EAT was 67.24%, and 19.61% of stations showed statistically significant increase (*p* < 0.05). In general, cumulative precipitation increased but sunshine hours decreased during soybean growing season. The probability of increased cumulative precipitation was 53.43%, with median change rates of 6.66 ± 0.93 mm decade^−1^. In addition, the increase rate of cumulative precipitation in Huang-Huai-Hai soybean zone (21.29 ± 2.33 mm decade^−1^) was much higher than those at southern soybean zone (11.90 ± 3.08 mm decade^−1^). In contrast, cumulative precipitation decreased by − 2.69 ± 0.80 mm decade^−1^ in northern soybean zone. Sunshine hours at 68.63% stations decreased and 33.33% stations showed statistically significant decrease (*p* < 0.05). Generally, the probability of decreased sunshine hours was 70.23% and the change rate was − 33.98 ± 1.05 h decade^−1^ nationally. Moreover, the decrease rate of sunshine hours during soybean growing seasons in southern soybean zone was the greatest (− 36.97 ± 1.90 h decade^−1^).

### Spatiotemporal changes in soybean phenological stages and key growth periods

Obvious changes in soybean’s phenological stages and key growth periods were distinguished over the study period. On a national scale, the SD (68.63% of stations), emergence date (52.94% of stations), trifoliate period (60.78% of stations), anthesis date (50.98% of stations), and podding date (58.82% of stations) of soybean were delayed, whereas the maturity date (58.82% of stations) was advanced (Fig. 3). Correspondingly, the delay of SD (1.02 ± 0.3 days decade^−1^) was the greatest, followed by emergence date (0.57 ± 0.29 days decade^−1^) and trifoliate period (0.55 ± 0.3 days decade^−1^). However, the probability of advanced maturity date was 57.44%, with a mean change rate of − 0.3 ± 0.23 days decade^−1^. As for changes in key growth periods, the vegetative growth period (60.78% of stations), and whole growth period (68.63% of stations) were shortened, whereas the reproductive growth period (49.02% of stations) was extended nationally (Fig. 4). The probability of shortened vegetative growth period, reproductive growth period and whole growth period were 54.46%, 52.13% and 63.05%, with median change rates of − 0.52 ± 0.24 days decade^−1^, 0.05 ± 0.26 days decade^−1^ and − 1.32 ± 0.3 days decade^−1^, respectively.

**Figure 2:**
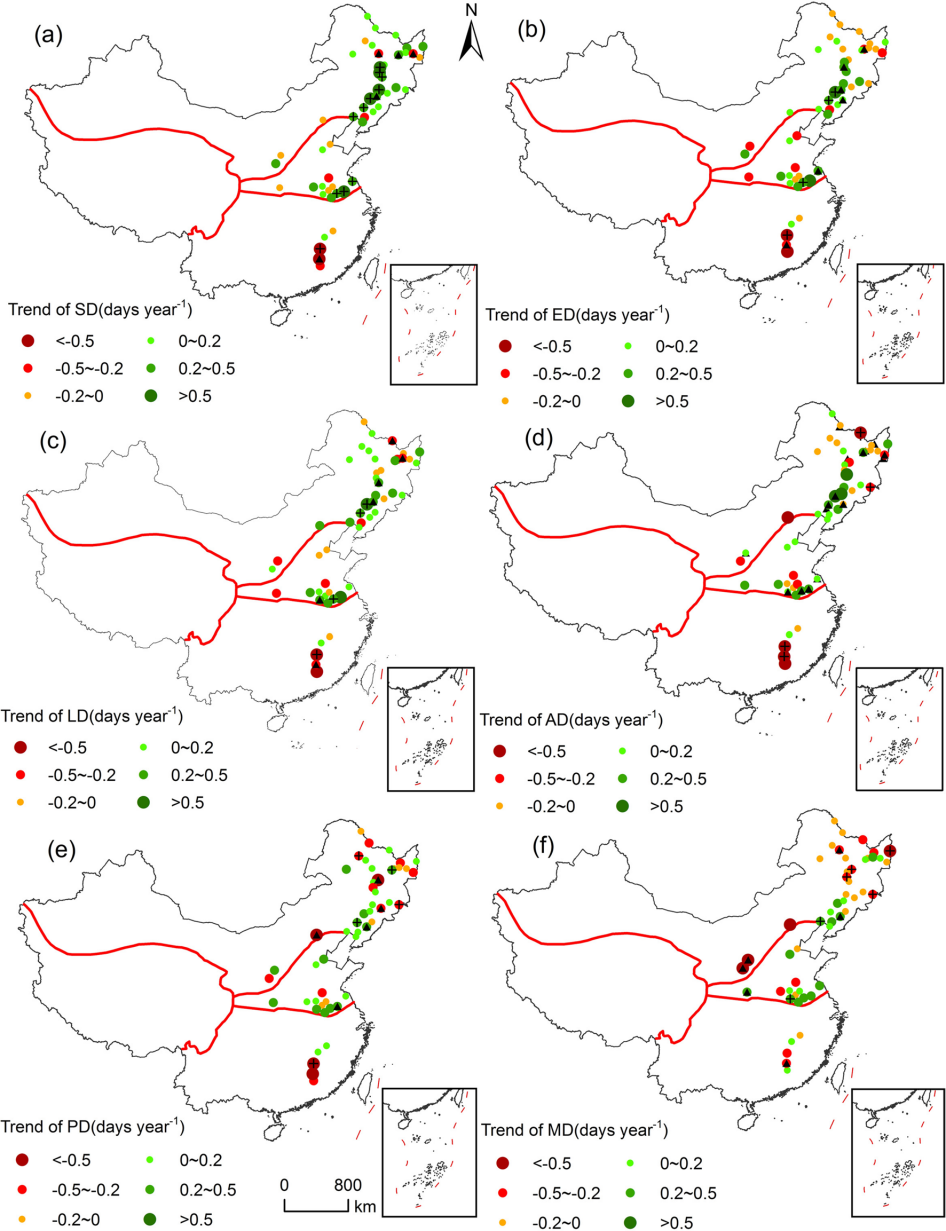
Distribution of different soybean phenological stage trends from 1992 to 2018 in China. (**a**)–(**f**) Represent SD, emergence date (ED), trifoliate period (LD), anthesis date (AD), podding date (PD), and maturity date (MD), respectively. The abbreviations are the same as in Fig. 1. The map was generated by ArcGIS 10.2 software (https://www.esri.com/).

**Figure 3:**
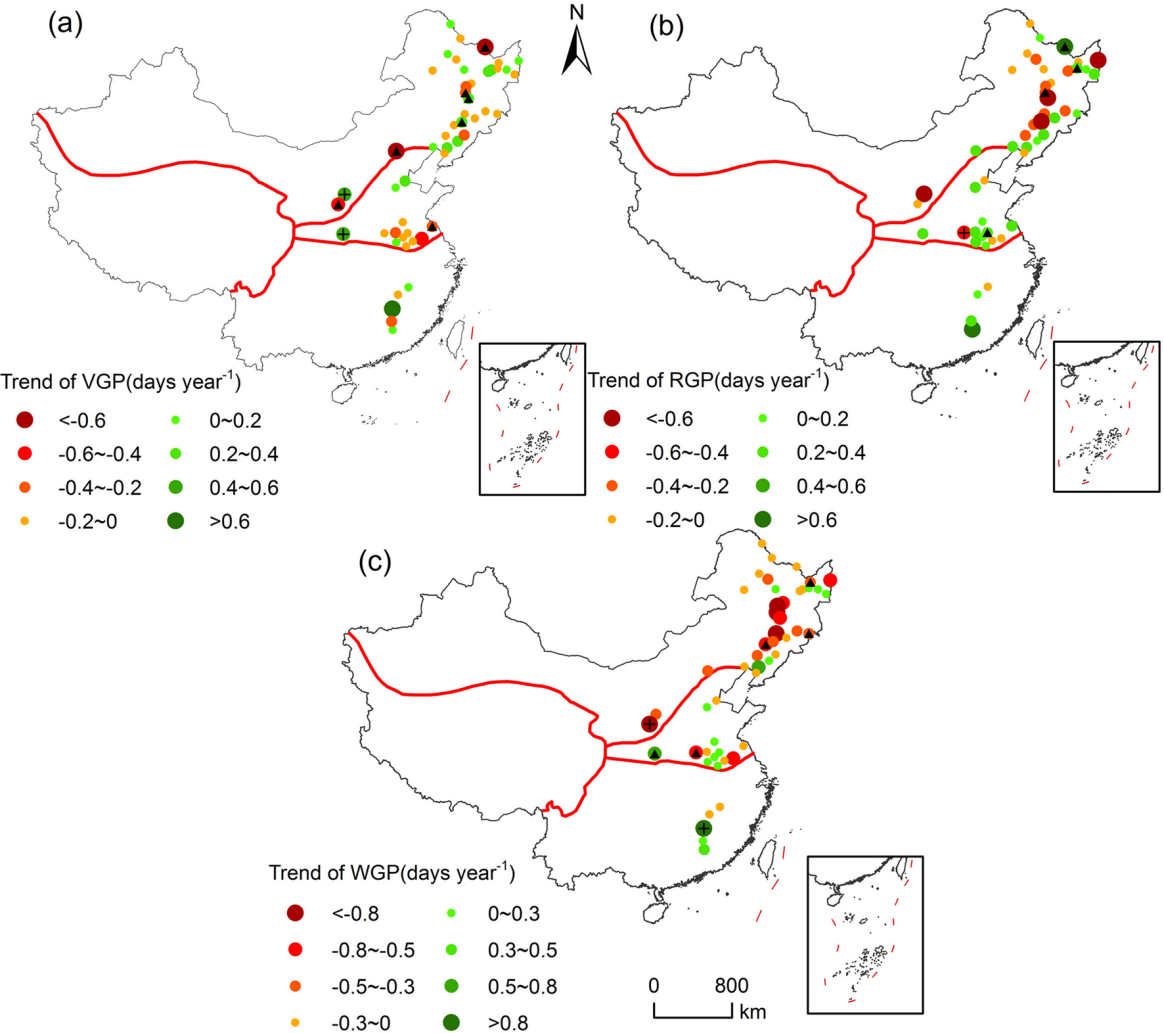
Changes in soybean’s growth periods from 1992 to 2018 in China. (**a**)–(**c**) Correspond to the whole growth period (WGP), vegetative growth period (VGP), and reproductive growth period (RGP), respectively. The map was generated by ArcGIS 10.2 software (https://www.esri.com/).

**Figure 4:**
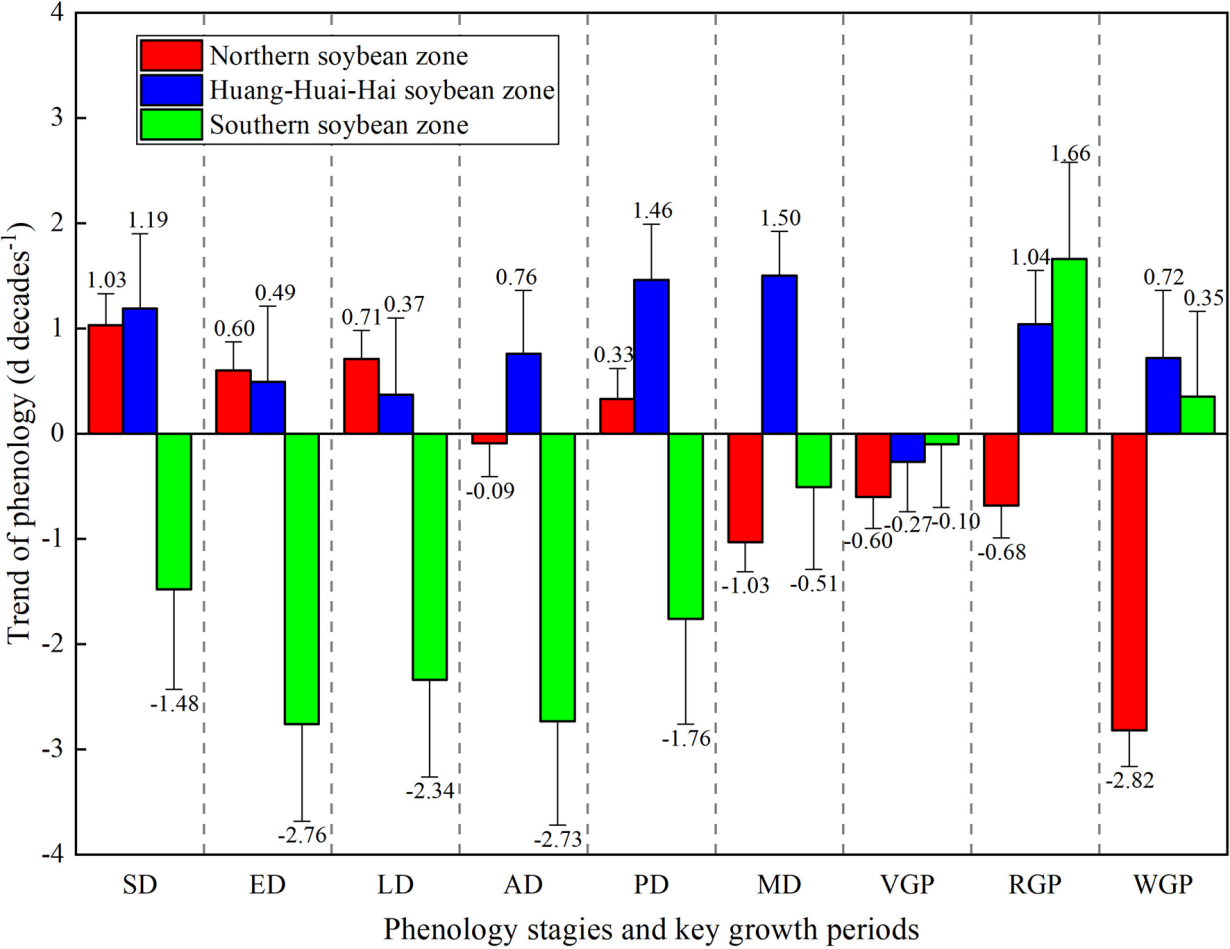
Changes in the phenology and key growth periods of soybeans under different cropping systems (numbers are the median value across stations in each soybean zone). The abbreviations are the same as in Figs. 2 and 3.

**Figure 5:**
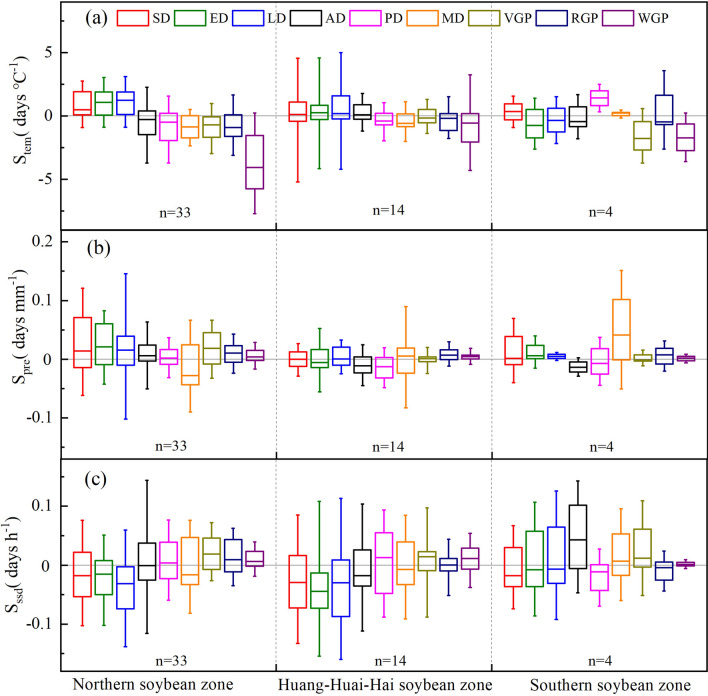
The sensitivity of soybean phenology to key climatic factors from 1992 to 2018 in China. The abbreviations are the same as in figures above.

The change trends of soybean phenological stages and key growth periods showed obvious difference across soybean cultivation regions (Figs. 2, 3). In northern soybean region, the SD, emergence date, trifoliate period, and podding date of soybean were delayed, whereas the anthesis date, and maturity date of soybean were advanced (Fig. 2). Among them, the delay of SD was the greatest, with change rate of 1.03 ± 0.3 days decade^−1^. In addition, more than 70% of stations showed advanced maturity date, and the median change rate was − 1.03 ± 0.28 days decade^−1^ in northern soybean zone (Fig. 2f). As shown in Fig. 4, key growth periods for spring soybean within the northern soybean zone were all shortened, with vegetative, reproductive, and whole growth period were shortened by 0.6 ± 0.3, 0.68 ± 0.31, and 2.82 ± 0.34 days decade^−1^, respectively. In the Huang-Huai-Hai soybean zone, the phenological stages of summer soybean were all delayed, and the median change rate of maturity date was the greatest (1.5 ± 0.42 days decade^−1^), followed by podding date (1.46 ± 0.53 days decade^−1^) and SD (1.19 ± 0.71 days decade^−1^). By contrast, the phenology of spring soybean was advanced at most stations in southern soybean zone, of which the advance of emergence date was the greatest (− 2.76 ± 0.92 days decade^−1^), followed by anthesis date (− 2.73 ± 0.99), and trifoliate period (− 2.34 ± 0.92 days decade^−1^). However, the changes of key growth period of soybean in the Huang-Huai-Hai soybean zone and northern soybean zone were consistent. On a national scale, the vegetative growth period was shortened, and the reproductive and the whole growth period were prolonged (Fig. 5). Among them, the median change rates of reproductive growth period were largest both in Huang-Huai-Hai soybean zone (1.04 ± 0.51 days decade^−1^) and northern soybean zone (1.66 ± 0.92 days decade^−1^). In addition, the trend of whole growth period in Huang-Huai-Hai soybean zone (0.72 ± 0.64 days decade^−1^) was more significant than that in northern soybean zone (0.35 ± 0.81 days decade^−1^). with an average rate of 5.60 days decade^−1^.

### Sensitivity of soybean phenological stages and key growth periods to different climatic factors

The soybean’s phenology and its key growth periods were sensitive to changes in key climatic factors (i.e., average temperature, cumulative precipitation, and cumulative sunshine hours), and exhibited obvious differences (Fig. 5). On average, the sensitivity of SD (74.51% stations), emergence date (70.59% stations), and trifoliate period (72.55% stations) to average temperature were positive, whereas those of anthesis date (56.86% stations), podding date (58.82% stations) and maturity date (66.67% stations) were negative. As 1 ℃ increase in average temperature, the SD, emergence date, and trifoliate period of soybean delayed by 0.37 ± 0.2, 0.68 ± 0.19, and 0.59 ± 0.2 days, while the anthesis date, podding date and maturity date were advanced by − 0.11 ± 0.19, − 0.29 ± 0.18, and − 0.6 ± 0.14 days, respectively (Fig. 5a). Moreover, the key growth periods of soybean were all negatively sensitive to average temperature. The negative temperature sensitivity of whole growth period (2.45 ± 0.24 days ℃^−1^) was greater than those of vegetative growth period (0.53 ± 0.16 days ℃^−1^), and reproductive growth period (0.71 ± 0.17 days ℃^−1^). Regionally, the SD, emergence date, and trifoliate period of soybean were positively correlated with average temperature, while the anthesis date, podding date and maturity date were negatively correlated with average temperature in the northern soybean zone. In addition, the trifoliate period strongly response to temperature change, and as 1 ℃ increase in average temperature, the trifoliate period delayed by 1.23 ± 0.2 days. In Huang-Huai-Hai soybean zone, the SD, emergence date, trifoliate period, and anthesis date of soybean were positively sensitive to temperature, whereas the podding date and maturity date were negatively sensitive. Among them, the sensitivity of maturity date to temperature was the greatest, with value of − 0.58 ± 0.28 days ℃^−1^. The SD, podding date and maturity date were advanced but the emergence date, trifoliate period, and anthesis date were delayed in southern soybean zone. Moreover, the podding date was most sensitive to temperature with the sensitivity value of 1.42 ± 0.42 days ℃^−1^. In addition, the key growth periods of soybean in three zones were all shortened by average temperature. The sensitivity of key growth periods to temperature in northern soybean zone and southern soybean zone was greater than that in Huang-Huai-hai soybean zone. Moreover, the sensitivity of whole growth period to temperature in the northern soybean zone was the greatest (− 4.05 ± 0.28 days ℃^−1^).

In the growing season, the sensitivity of soybean phenology and key growth periods to precipitation and sunshine time varies greatly. (Fig. 5b,c). On average, the SD (54.90% stations), emergence date (58.86% stations), trifoliate period (64.71% stations), and anthesis date (54.90% stations) were positively sensitive to precipitation while the podding date (50.98% stations) and maturity date (52.94% stations) were negatively sensitive. The sensitivity of emergence date to precipitation was the greatest, with median value of − 0.01 days (10 mm)^−1^. The key growth periods were prolonged by precipitation nationally, and the sensitivity of reproductive growth period was largest (0.10 ± 0.02 days (10 mm)^−1^). Except for maturity date, the remained phenology stages and key growth periods were positively sensitive to precipitation in northern soybean zone. Similarly, key growth periods of soybean in Huang-Huai-Hai soybean zone were also prolonged as precipitation increases. However, the sensitivity of emergence date, anthesis date and podding date to precipitation were negative in Huang-Huai-Hai soybean zone. In the southern soybean area, with the increase of precipitation, the reproductive growth period and the entire growth period were prolonged, but the vegetative growth period was shortened. Except for podding date, the sensitivity of other phenology stages to sunshine hours were negative in general. Moreover, the key growth periods were extended with the increase of sunshine hours. The SD, emergence date, and trifoliate period of soybean were all negatively sensitive to sunshine hours in three zones, and the median sensitivity values in Huang-Huai-Hai soybean zone were greater than those in both northern soybean zone and southern soybean zone. The vegetative growth period and whole growth period were prolonged as sunshine hours increased in three soybean cultivation zones. The sensitivity value of vegetative growth period was the greatest (0.19 ± 0.10 day (10 h)^−1^). By contrast, the sensitivity of reproductive growth period to sunshine hours was negative in southern soybean zone.

## Discussion

### Spatial variation of soybean phenology and their sensitivity to climatic factors

Revealing the spatial pattern of changes in soybean phenology is critical for guiding regional agricultural activities in attempts to mitigate the negative impacts of climate change and ensure stable soybean production^32,33^. However, only a few have attempted to detect the spatiotemporal pattern of soybean phenology, focusing on some limited key phenological stages (mainly datesf anthesis or maturity). This study revealed that the observed dates of sowing, emergence, and anthesis were delayed, while the vegetative growth period and whole growth period were shortened on a national scale. Such a changing pattern in growing phase was consistent with the study of soybean in eastern China^34^. However, the changes in multiple consecutive soybean phenological stages and key growth periods showed obvious spatial heterogeneity. The soybean phenological stages mostly delayed in Huang-Huai-Hai soybean zone whereas advanced in southern soybean zone. Simultaneously, the key growth periods were shortened in northern soybean zone while the vegetative growth period and whole growth period were prolonged in Huang-Huai-Hai zone and southern soybean zone. Moreover, the degree of changes in soybean phenology also differed in regions. For instance, the delays of SD and podding date in Huang-Huai-Hai soybean zone were greater than those in northern soybean zone. However, the delay of emergence date and trifoliate period in those two zones showed opposite pattern (Fig. 4).

The regional differences in the phenological trends of China’s main food crops have attracted widespread attention, and most studies have explored the key influencing factors^12,18,21^. Climate warming was identified as one of the most decisive factors causing changes in crop phenology^32^. As shown in Fig. 5a, the key growth periods of soybean were negatively sensitive to the increase in average temperature during the soybean growing season. This indicates that as the temperature increases, the key growth period is shortened, consistent with previous findings that increase in temperature will accelerate the growth rate of crops, promote crop phenology, and shorten the key growth period^31,35^. Moreover, the response of soybean phenology to temperature rise varied in regions. The sensitivity of anthesis date and podding date to average temperature within the southern soybean zone was much higher than those in the northern and Huang-Huai-Hai soybean zones, indicating that soybean yield formation in low latitude regions is more sensitive to temperature. Appropriate soil moisture, temperature and sunshine hours are all necessary conditions for seed germination^36^. The SD was significantly delayed in the northern soybean area. However, the delay of SD may lead to insufficient sunshine hours, affecting the emergence of soybean in high-latitude northern soybean zone. In contrast, owing to the relatively dry climate during summer soybean-sowing period within the Huang-Huai-Hai zone, the increase in precipitation will effectively supplement soil moisture before and after planting, which may alleviate drought stress during the SD period and promote the emergence of soybean. In addition, sunshine hour is a key climatic factor affecting the growth and development of soybean. Our results showed that soybean phenological stages were mostly advanced in northern soybean zone and Huang-Huai-Hai soybean zone by increasing sunshine hours. The main reason was that as the high-latitude northern soybean zone experiences relatively lower temperatures, extra heat and light owing to more sunshine hours can effectively meet the soybean’s growth photothermal demands and thus accelerate crop development^37^. Since soybeans are short-term plants^38^, the sunshine hours in the southern soybean region with sufficient light and heat resources has increased, whereas the soybean podding period is delayed, and the reproductive growth period is shortened, which may hinder the formation of soybean yield. Thus, it is noteworthy that the impact of sunshine hours on soybean phenology varied in different regions, especially the negative sensitivity of reproductive growth period to increase in cumulative sunshine hours in southern soybean zone. Most of the process-based models previously recommended for soybean development and yield simulation only consider the effect of sunshine hours on the flower induction period^39^. The results presented here instead suggest that it is necessary to evaluate the impact of sunshine hours on different growth periods during soybean development to improve the accuracy of growth and yield simulations in the future.

### Effects of phenological changes on soybean yield and adaptation measures to climate change

The changes in soybean phenology and corresponding climate conditions significantly affect the soybean yield formation^40,41^. Up to date, there is lack of studies exploring the impact of soybean phenological changes on yield at regional and national scale, except some field experiments conducted at site scale in China^42^. Consistent to our results, previous studies showed that the anthesis stage of soybean was advanced and the length of whole growth period was shortened in the context of climate warming, which decreased the leaf photosynthetic rate and accumulation of dry material, leading to decrease of 100-seed weight and soybean yield in North China Plain and southern China^42,43^. However, the yield of newly-approved soybean varieties with a prolonged growth duration has increased significantly in Northeast China^26^. These regional inconsistencies indicated that variation of changes in soybean phenology in the context of climate change could lead to distinct regional variation of yield changes. Further study on the impact of soybean phenological changes on the final yield in different regions is needed. On the basis, specific regional adaptation measures should be taken accordingly to ensure the safety of soybean production.

SD shifts has been proven to be one of the main anthropogenic management measures affecting crop phenological changes^44,45^. Any SD shift will affect the time and length of soybean development to a certain extent^22,46^, and shifting SD within an optimum planting range in different regions could mitigate the adverse impacts of climate conditions on soybean yield^47^. The results showed that, in the context of climate change, the delay in SD was accelerated by the increase in average temperature in three soybean cultivation zones. This suggests that SD adjustments can change temperature conditions throughout the soybean growing season, making it possible to improve the utilization efficiency of heat resources throughout the growing season^19^. Similarly, SD shifts is also influenced by changes in precipitation and sunshine hours throughout the reproductive growth period. Our results showed that the advancement of soybean SD slowed down the decline of precipitation, and the delay of SD can slow down the growth trend of sunshine time throughout the reproductive growth period. Therefore, shifting SD can be used to adjust the environment for soybean growth and maximize the utilization efficiency of water and fertilizer resources^48^. This approach provides an effective management measure that enables soybean farming to adapt to climate change.

In addition to adjustments in sowing dates, cultivar replacement affects the phenological changes and yield formation of soybean. Breeding and cultivar improvement are considered as the most effective measures for increasing soybean yields^49^. Based on statistics collected from 29 stations containing variety records, soybean varieties were replaced more than five times during the study period, which could have affected the phenology and yield formation of soybean. Tao et al.^50^ found crop cultivars’ thermal requirements to complete each single development stage changed differently. In our study, increasing temperature and EAT during the soybean growing season did not significantly advance soybean phenological stages but shortened key growth periods in northern soybean zone. This may due to the climate warming within the range suitable for soybean growth, which is likely to increase soybean production. Therefore, new cultivars with longer vegetative, reproductive, and seed filling periods will be favorable to capitalize on the extra heat in northern soybean zone^46^. By contrast, key growth periods of soybean were extended at most stations in Huang-Huai-Hai soybean zone and southern soybean zone, which may increase the heat waves during the growing periods in low-latitude regions. To mitigate the adverse impact of increasing temperature, cultivating soybean variety with heat-resistance characteristic or high thermal demand will be effectively decrease the temperature sensitivity of soybean growth and yield^34,44^. In addition, uneven precipitation in the future will result in dry or wet soil, which will lead to soybean yield reduction, especially drought in flowering and podding periods^51^. In northern soybean region, the cumulative precipitation during soybean growing period was decreased, which could negatively affected soybean yield with synchronous warming. Sowing drought-tolerant varieties and appropriate increase of irrigation during the vegetative growing period can help alleviate the drought in this region. Moreover, genetic improvement in soybean cultivars with decreasing photo-thermal sensitivity is expected as the sunshine hours generally decreased during the soybean growing period.

### Uncertainties

We carried out the study using the dataset (1992–2018) of phenological and meteorological data collected at 51 stations across China. There are differences between results of our research and previous studies^26,34^, which may owe to the difference in study period and research stations as well as regions. Another possible uncertainty of this study may stem from the statistical method we used. Changes in soybean phenological stages and key growth periods were assumed as a constant or linear response to climate change. Three key climatic factors (namely temperature, precipitation and sunshine hours) were considered. Actually, the impact of climate change on crop growth and yield is complex, and there are interactions between climatic factors^52^, which also increases the uncertainty of the results. Moreover, the frequency and intensity of climate extreme events such as heat stress and extreme drought increased, which are detrimental to the growth of crop and may directly lead to the death of crops^11,51^. In addition, other climatic factors such as increased CO_2_ concentration, which can cause a ‘fertilization effect’ and increase crop biomass^53^, also have a noteworthy influence on crop growth. Crop growth and yield are co-determined by the interaction between genotypes, environment, and management practices^54^. Different soybean varieties and growth environments like soil properties, and management measures (i.e., irrigation and fertilization) can affect the growth of soybean. All of these factors can incur a sharp shift of soybean phenology, therefore, combined and discriminate impact of climate change and management measures on soybean phenology is a topic that we will address in the future.

## Conclusion

This study assessed the trends of soybean phenological stages and key growth periods, and evaluated the sensitivity soybean to climatic factors (i.e. average temperature, cumulative precipitation, and cumulative sunshine hours) from 1992 to 2018 in China. Results showed that (1) during the soybean growing season, the average temperatures, EAT, and cumulative precipitation remarkedly increased but cumulative sunshine hours decreased. The change rates of climatic factors varied in regions. (2) The soybean phenological stages were mostly delayed and key growth periods were shortened on a national scale, but soybean phenology changed to different extent in regions. The key growth periods of soybean were shortened, and the change trend of whole growth period was the greatest (− 2.82 ± 0.34 day decades^−1^) in northern soybean zone. (3) Increasing temperature was the most critical climatic factor to soybean phenological changes, advancing soybean phenological stages and shortening key growth periods. Overall, the sensitivity of soybean key growth periods to cumulative precipitation and sunshine hours was positive. Moreover, phenological stages at most stations were negatively sensitive to cumulative sunshine hours. Crop management measures such as sowing date adjustment and variety replacement can be used for regulating soybean growth and climate change adaptation in China.

## Materials and methods

### Study area

**Figure 6:**
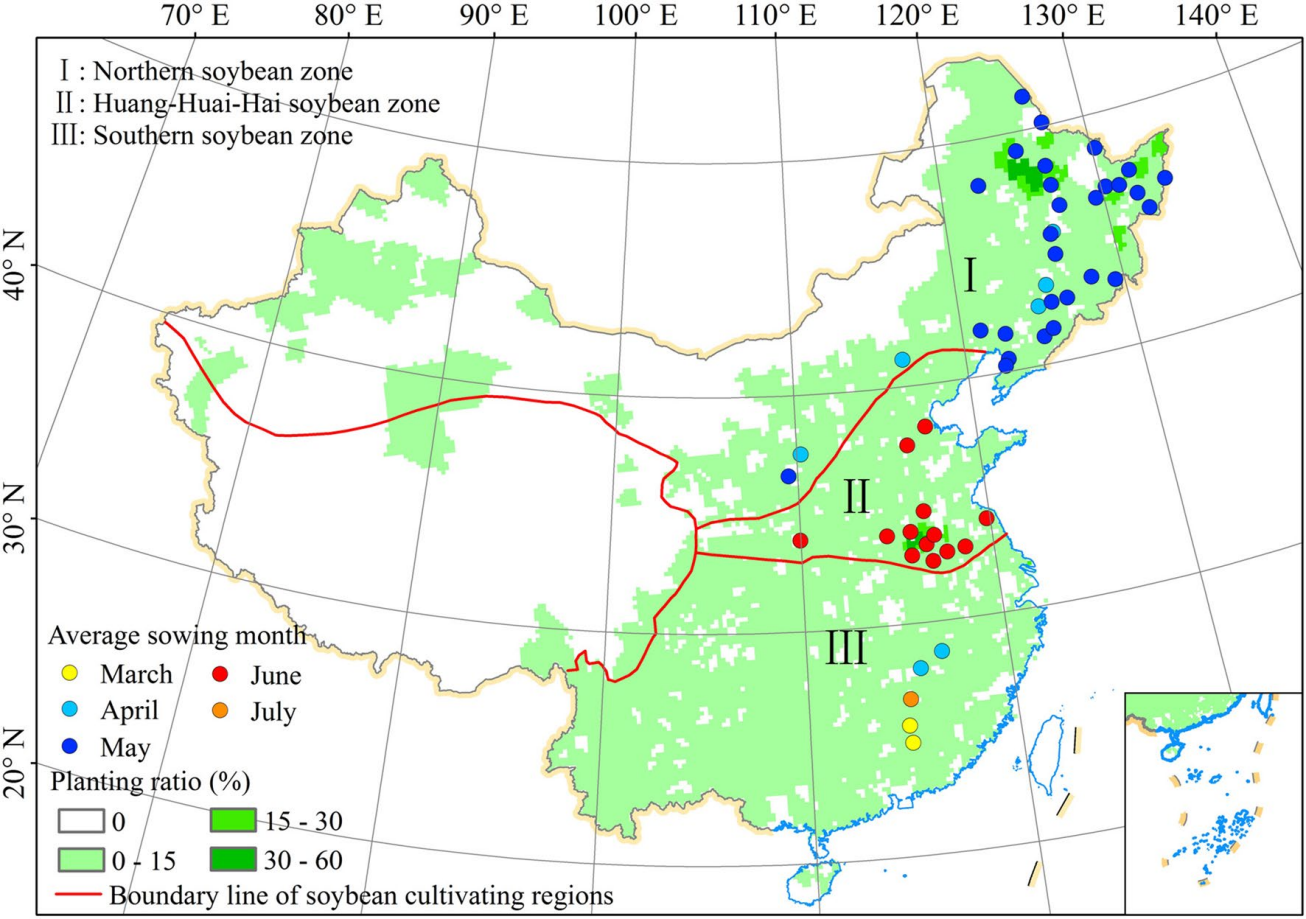
Spatial distribution of three soybean cultivation regions and 51 agro-meteorological stations in China. The map was generated by ArcGIS 10.2 software (https://www.esri.com/).

The spatial distribution of 51 agro-meteorological stations and the soybean cultivation divisions were shown in Fig. 6. The entire soybean-planting area in China is divided into three major regions: the northern soybean zone, the Huang-Huai-Hai soybean zone, and the southern soybean zone^55,56^. Phenology data of soybean and corresponding meteorological data at 51 stations during the period of 1992 and 2018 were collected from the China Meteorological Data website (http://data.cma.cn/site/index.html). The phenological data covered the dates of the six phenological stages, namely SD, emergence date, trifoliate period, anthesis date, podding date, and maturity date. The key growth periods defined in this study comprised vegetative growth period (emergence-to-anthesis), reproductive growth period (anthesis-to-maturity), and whole growth period (sowing-to-maturity). In addition, the climatic factors including daily average temperature, cumulative precipitation, and sunshine hours during soybean growing season were calculated as average temperature, cumulative precipitation and cumulative sunshine hours. The 51 soybean stations in the study were subdivided into 33 stations in the northern soybean zone, 14 stations in the Huang-Huai-Hai soybean zone, and 4 stations in the southern soybean zone on the basis of the SDs, ripening times, and regional differences. The corresponding soybean types included in this analysis consisted of northern spring, summer, and southern spring soybeans. The growing season information including sowing month, maturity month and average growing season length of each station were listed in Table S1. Generally, the spring soybean in northern spring soybean zone was sown in late April or early May and matured in September, summer soybean in the Huang-Huai-Hai soybean zone was sown in late May and early June and matured in September or October, and the spring soybean in the southern spring soybean zone was sown in March and April, and matured in June or July of each year (Table S1).

### Changes in phenology, key growth periods and corresponding climatic factors

The linear regression method was used to calculate the change trends in soybean phenology and corresponding climatic factors within relevant periods. A linear regression model was established using year as the independent variable and the date of soybean phenology appearance or the length of soybean growth period as the dependent variables. Corresponding periods of climatic factors were expressed as monthly averages or cumulation, in which a given corresponding soybean phenological event has occurred. For example, the corresponding SD period was defined as the month in which average SD occurs, while the corresponding vegetative growth period was defined as starting from the month containing the average emergence date to the month containing the average anthesis date. Thus, trends in soybean phenology and growth periods at selected stations were calculated as follows:

1 $$Y_{phe} = S_{phe} \times Year + I_{phe},$$

where $Y_{phe}$ denotes the soybean phenology dates or growth period length (days), *Year* denotes the year, $I_{phe}$ denotes intercept, and $S_{phe}$ denotes the slope of the regression equation, equivalent to changes in the corresponding phenological period or growth period length (days a^−1^). Change trends in climatic factors were calculated as follows:

2 $$Y_{cli} = S_{cli} \times Year + I_{cli} ,$$

where $Y_{cli}$ denotes temperature (°C), precipitation (mm), or sunshine hours (h) for a corresponding observation period, while *Year* denotes the year,${ }I_{cli}$ denotes the intercept, and $T_{cli}$ denotes the slope of the regression equation, equaling the change trend of corresponding climatic factors. A paired t-test was used to evaluate the statistical significance of change trends at each station.

### Sensitivity of soybean phenological stages and key growth periods to climatic factors

To analyze the sensitivity of soybean phenological stages and key growth periods to the major climatic factors, a multiple regression model was built based on observed data bearing on these variables, as follows:

3 $$Phe = S_{tem} \times Tem + S_{pre} \times Pre + S_{ssd} \times Ssd + int,$$

where $Phe$ denotes soybean phenological dates or growth period lengths (days) observed at planting stations; $Tem$,$Pre$, and $Ssd$ respectively denote the temperature, precipitation, and sunshine hours for corresponding periods; $int$ denotes the fitted equation intercept; and $S_{tem} , S_{pre} ,{\text{and }}S_{ssd}$ respectively denote temperature (days ℃^−1^), precipitation (days mm^−1^), and sunshine hours coefficients (days h^−1^) in soybean’s phenology or growth periods.

### Calculation of effective accumulated temperature (EAT) during soybean phenological stages and key growth periods

EAT is an important indicator used to evaluate the heat required for crop growth and development. The EAT during soybean growing season used here represents the cumulative daily temperature value which exceeds the basic temperature for crop development during a corresponding soybean phenological period. This variable was calculated as follows:

4 $$EAT = \mathop \sum \limits_{1}^{n} \left( {T_{mean} - T_{base} } \right),$$

where EAT denotes the effective accumulated temperature during a given phenological or growth period; *n* denotes the number of days within each corresponding period; $T_{mean}$ denotes daily temperature, and $T_{base}$ is the basic soybean development temperature (= 10℃ in this analysis).

### Analysis of uncertainty

Cumulative distribution functions were used to analyze the uncertainties of the changes in soybean phenology, key growth periods and climate factors during the soybean growing seasons. The cumulative probability for change rate < 0, represents the cumulative probability of advanced phenological stages, shortened key growth periods or decreased climate factors.

## Supplementary Information

Supplementary Information

## Data Availability

All data generated or analyzed during this study are included in this published article.
