# Supplementary material for: Shortened key growth periods of soybean observed in China under climate change
Source: Sci Rep. 2021 Apr 14;11:8197. doi: 10.1038/s41598-021-87618-9 (PMC8047036; doi:10.1038/s41598-021-87618-9)
Supplement: Supplementary file 1 — Supplementary Information [file 41598_2021_87618_MOESM1_ESM.docx]

**Shortened key growth periods of soybean observed in China under climate change**

Yujie Liu^1,2*^, Qinghua Tan^1,2^, Liang Dai^1,2^

1. Key Laboratory of Land Surface Pattern and Simulation, Institute of Geographic Sciences and Natural Resources Research, Chinese Academy of Sciences, Beijing 100101, China;

2. University of Chinese Academy of Sciences, Beijing, 100049, China

*Corresponding Author: Yujie Liu([liuyujie@igsnrr.ac.cn](mailto:liuyujie@igsnrr.ac.cn))

| Supplementary table Table S1. Geographical and cultivation information of soybean at 51 stations in China. | | | | | | | | | | |
| --- | --- | --- | --- | --- | --- | --- | --- | --- | --- | --- |
| Cultivation zone | Soybean types | Station number | Longtitude (˚) | Latitude (˚) | Altitude (m) | Study period (year) | Sowing month | Maturity month | Average growing season length(d) |  |
| Northern soybean zone | spring soybean | 50867 | 127.21 | 46.50 | 134.10 | 1992-2018 | May | Sep-Oct | 134±8 |  |
| Northern soybean zone | spring soybean | 50888 | 132.11 | 46.19 | 79.60 | 1992-2018 | May | Sep-Oct | 130±8 |  |
| Northern soybean zone | spring soybean | 54349 | 124.70 | 41.18 | 258.90 | 1992-2011 | May | Sep-Oct | 144±6 |  |
| Northern soybean zone | spring soybean | 54243 | 124.70 | 42.47 | 165.10 | 1992-2011 | Apr | Sep-Oct | 135±9 |  |
| Northern soybean zone | spring soybean | 50655 | 126.90 | 48.28 | 526.10 | 1992-2018 | May | Sep | 137±7 |  |
| Northern soybean zone | spring soybean | 54186 | 128.12 | 43.22 | 735.10 | 1992-2018 | May | Sep-Oct | 143±1 |  |
| Northern soybean zone | spring soybean | 54308 | 116.38 | 41.13 | 62.70 | 1992-2011 | Apr | Sep-Oct | 145±6 |  |
| Northern soybean zone | spring soybean | 50787 | 131.98 | 47.23 | 24.80 | 1992-2011 | May | Sep-Oct | 131±8 |  |
| Northern soybean zone | spring soybean | 54474 | 122.21 | 40.25 | 118.30 | 1992-2011 | May | Sep | 134±9 |  |
| Northern soybean zone | spring soybean | 50953 | 126.46 | 45.45 | 25.30 | 1992-2018 | May | Sep-Oct | 130±1 |  |
| Northern soybean zone | spring soybean | 54472 | 122.43 | 40.53 | 247.40 | 1992-2018 | May | Sep | 140±4 |  |
| Northern soybean zone | spring soybean | 50756 | 126.97 | 47.43 | 166.40 | 1992-2018 | May | Sep-Oct | 127±5 |  |
| Northern soybean zone | spring soybean | 50468 | 127.27 | 50.15 | 173.90 | 1992-2018 | May | Sep-Oct | 132±8 |  |
| Northern soybean zone | spring soybean | 50353 | 126.39 | 51.43 | 100.20 | 1992-2018 | May | Sep | 124±8 |  |
| Northern soybean zone | spring soybean | 50983 | 132.58 | 45.46 | 263.22 | 1992-2018 | May | Sep | 132±1 |  |
| Northern soybean zone | spring soybean | 54273 | 126.45 | 42.59 | 102.30 | 1992-2018 | May | Sep | 144±8 |  |
| Northern soybean zone | spring soybean | 50880 | 131.13 | 46.72 | 82.00 | 1992-2018 | May | Sep-Oct | 135±1 |  |
| Northern soybean zone | spring soybean | 50873 | 130.28 | 46.82 | 90.40 | 1992-2018 | May | Sep-Oct | 132±9 |  |
| Northern soybean zone | spring soybean | 50673 | 130.24 | 48.53 | 65.90 | 1992-2018 | May | Sep-Oct | 130±7 |  |
| Northern soybean zone | spring soybean | 54337 | 121.10 | 41.90 | 253.00 | 1992-2018 | May | Sep | 138±7 |  |
| Northern soybean zone | spring soybean | 54260 | 125.50 | 42.55 | 242.20 | 1992-2018 | May | Sep-Oct | 148±7 |  |
| Northern soybean zone | spring soybean | 50557 | 125.23 | 49.17 | 54.40 | 1992-2018 | May | Sep-Oct | 132±7 |  |
| Northern soybean zone | spring soybean | 50892 | 134.00 | 46.48 | 168.30 | 1992-2018 | May | Sep-Oct | 131±7 |  |
| Northern soybean zone | spring soybean | 50955 | 126.30 | 45.38 | 218.80 | 1992-2018 | May | Sep-Oct | 144±1 |  |
| Northern soybean zone | spring soybean | 54165 | 125.39 | 43.30 | 929.70 | 1992-2018 | May | Sep-Oct | 117±9 |  |
| Northern soybean zone | spring soybean | 53754 | 110.22 | 37.50 | 95.10 | 1992-2018 | Apr | Oct | 165±1 |  |
| Northern soybean zone | spring soybean | 50871 | 129.53 | 46.44 | 266.80 | 1992-2018 | May | Sep-Oct | 135±9 |  |
| Northern soybean zone | spring soybean | 54353 | 125.30 | 41.44 | 328.40 | 1992-2018 | May | Sep | 150±5 |  |
| Northern soybean zone | spring soybean | 54333 | 122.50 | 41.59 | 30.90 | 1992-2018 | May | Sep-Oct | 155±7 |  |
| Northern soybean zone | spring soybean | 53845 | 109.50 | 36.60 | 1180.50 | 1992-2018 | May | Sep-Oct | 140±9 |  |
| Northern soybean zone | spring soybean | 54292 | 129.47 | 42.88 | 258.00 | 1992-2018 | May | Sep-Oct | 144±4 |  |
| Northern soybean zone | spring soybean | 54072 | 126.32 | 44.50 | 196.50 | 1992-2018 | Apr | Sep | 154±1 |  |
| Northern soybean zone | spring soybean | 50639 | 122.44 | 48.00 | 306.50 | 1992-2018 | May | Sep-Oct | 129±9 |  |
| Huang-Huai-Hai soybean zone | summer soybean | 58049 | 119.82 | 34.03 | 4.10 | 1992-2018 | Jun | Sep-Oct | 121±1 |  |
| Huang-Huai-Hai soybean zone | summer soybean | 58102 | 115.77 | 33.87 | 39.10 | 1992-2018 | Jun | Sep-Oct | 102±6 |  |
| Huang-Huai-Hai soybean zone | summer soybean | 58012 | 116.58 | 34.68 | 42.00 | 1992-2005, 2008-2011 | Jun | Sep-Oct | 106±5 |  |
| Huang-Huai-Hai soybean zone | summer soybean | 58222 | 117.55 | 32.87 | 24.60 | 1992-2018 | Jun | Sep-Oct | 99±5 |  |
| Huang-Huai-Hai soybean zone | summer soybean | 54710 | 116.10 | 37.52 | 18.60 | 1992-2018 | Jun | Sep-Oct | 127±7 |  |
| Huang-Huai-Hai soybean zone | summer soybean | 58203 | 115.73 | 32.87 | 32.70 | 1992-2018 | Jun | Sep-Oct | 95±6 |  |
| Huang-Huai-Hai soybean zone | summer soybean | 54624 | 117.21 | 38.22 | 4.50 | 1992-2018 | Jun | Sep-Oct | 100±8 |  |
| Huang-Huai-Hai soybean zone | summer soybean | 58118 | 116.53 | 33.28 | 26.00 | 1992-2018 | Jun | Sep-Oct | 101±5 |  |
| Huang-Huai-Hai soybean zone | summer soybean | 57143 | 109.97 | 33.87 | 747.20 | 1992-2018 | Jun | Sep-Oct | 102±8 |  |
| Huang-Huai-Hai soybean zone | summer soybean | 58215 | 116.78 | 32.55 | 25.70 | 1992-2011 | Jun | Sep-Oct | 99±6 |  |
| Huang-Huai-Hai soybean zone | summer soybean | 57899 | 114.92 | 26.80 | 71.40 | 1992-2011 | May | Oct | 100±8 |  |
| Huang-Huai-Hai soybean zone | summer soybean | 57193 | 114.52 | 33.78 | 52.60 | 1992-2011 | Jun | Sep-Oct | 102±8 |  |
| Huang-Huai-Hai soybean zone | summer soybean | 58122 | 116.98 | 33.63 | 25.70 | 1992-2018 | Jun | Sep-Oct | 96±7 |  |
| Huang-Huai-Hai soybean zone | summer soybean | 58138 | 118.52 | 32.98 | 40.80 | 1992-2018 | Jun | Sep | 91±5 |  |
| Southern soybean zone | spring soybean | 59092 | 114.82 | 24.92 | 250.20 | 1992-2011 | Mar | Jun | 107±6 |  |
| Southern soybean zone | spring soybean | 57992 | 114.75 | 25.67 | 127.00 | 1992-2011 | Mar | Jun-Jul | 100±4 |  |
| Southern soybean zone | spring soybean | 58612 | 116.68 | 28.70 | 21.10 | 1992-2018 | Apr | Jul | 99±7 |  |
| Southern soybean zone | spring soybean | 58608 | 115.55 | 28.07 | 66.20 | 1992-2018 | Apr | Jul | 108±8 |  |
